# Human Intervention Study to Assess the Effects of Supplementation with Olive Leaf Extract on Peripheral Blood Mononuclear Cell Gene Expression

**DOI:** 10.3390/ijms17122019

**Published:** 2016-12-02

**Authors:** Anna Boss, Chi Hsiu-Juei Kao, Pamela M. Murray, Gareth Marlow, Matthew P. G. Barnett, Lynnette R. Ferguson

**Affiliations:** 1 Discipline of Nutrition and Dietetics, The University of Auckland, Auckland 1142, New Zealand; abos517@aucklanduni.ac.nz (A.B.); ben.kao@auckland.ac.nz (C.H.-J.K.); 2 Auckland Cancer Research Society, Faculty of Medical and Health Sciences, The University of Auckland, Auckland 1142, New Zealand; p.murray@auckland.ac.nz; 3 Institute of Medical Genetics, UHW Main Building, Cardiff University, Cardiff CF10 3XQ, UK; MarlowG@cardiff.ac.uk; 4 Food Nutrition & Health Team, Food & Bio-Based Products Group, AgResearch Limited, Palmerston North 4442, New Zealand; matthew.barnett@agresearch.co.nz

**Keywords:** polyphenols, transcriptomics, nutraceutical, inflammation

## Abstract

Olive leaf extract (OLE) has been used for many years for its putative health benefits, but, to date, scientific evidence for the basis of these effects has been weak. Although recent literature has described a link between ailments such as cardiovascular disease, diabetes and cancer and a protective effect of polyphenols in the OLE, the mode of action is still unclear. Here, we describe a double-blinded placebo (PBO)-controlled trial, in which gene expression profiles of peripheral blood mononuclear cells from healthy male volunteers (*n* = 29) were analysed to identify genes that responded to OLE, following an eight-week intervention with 20 mL daily consumption of either OLE or PBO. Differences between groups were determined using an adjusted linear model. Subsequent analyses indicated downregulation of genes important in inflammatory pathways, lipid metabolism and cancer as a result of OLE consumption. Gene expression was verified by real-time PCR for three genes (*EGR1*, *COX-2* and *ID3*). The results presented here suggest that OLE consumption may result in health benefits through influencing the expression of genes in inflammatory and metabolic pathways. Future studies with a larger study group, including male and female participants, looking into direct effects of OLE on lipid metabolism and inflammation are warranted.

## 1. Introduction

Although olive leaf extract (OLE) has been used for many years for its putative health benefits, the mode of action by which these benefits occur is still unclear. In vitro and animal studies suggest that the health benefits of OLE are due, at least in part, to OLE polyphenols influencing gene expression [1,2]. In vitro studies using human cell lines have suggested that OLE has anti-inflammatory [3] and anti-cancer effects [2]. In vivo animal studies suggest that OLE attenuates obesity from a high fat diet [4] and reduces impacts resulting from inflammatory cytokine production and insulin resistance [5]. These studies showed altered expression of genes in relevant pathways. Animal [6] and in vitro [7] models have pointed towards interactions with Nrf2 (Nuclear factor erythroid 2-related factor 2) signalling cascades as one effector for the health benefits of polyphenols. However, this hypothesis has not been substantiated in human trials [8].

Recent research has shown improved blood pressure in hypertensive males with OLE [9] and improved insulin sensitivity in overweight males [10]. These studies have focused on specific outcomes or biomarkers such as improved insulin sensitivity or the expression of specific cytokines; however, a more comprehensive analysis of the changes in gene expression has not been undertaken.

Investigating changes in gene expression profile after OLE consumption in humans will enable a better understanding of the pathways involved. The genetic pathways and relationships uncovered can be used to hypothesise the modes of action. This may help substantiate existing health benefits and lead to the discovery of novel health benefits that may arise from the consumption of OLE.

This intervention study was focussed on identifying the genes and associated pathways that respond to OLE treatment and to determine the underlying mechanisms that correlate to reported health benefits. The tested product is designed for chronic consumption as a nutraceutical to improve health in the long term, as opposed to medicines that are developed for short-term use to create maximum results. A modest change over a long time is likely to manipulate health significantly [11]. Because the participants were healthy men, inflammatory and disease markers were in the healthy range; therefore, reducing these is difficult to achieve. The results are therefore intended to be indicative and to reveal important directions for future research.

## 2. Results

### 2.1. Participant Characteristics and Compliance, and Supplement Tolerance

A total of 32 men gave written consent and were enrolled in the study. Two participants were lost to follow-up and one participant withdrew due to an adverse reaction to the supplement. The first participants began the intervention on the 3 May 2015 and the last participants began on the 22 June 2015. The intervention ran for eight weeks.

Characteristics of the final 29 men (15 in the OLE arm and 14 in the placebo (PBO) arm of the intervention) are shown in Appendix Table A1. There were no statistically significant differences between intervention arms, or between the beginning and end of the intervention, for any of the measured characteristics.

On average, participants taking the OLE did not achieve the instructed 20 mL per day, most likely due to its bitter taste. However, the amount they did consume (~17 mL) means that their daily phenolic intake was higher than that approved by European Food Safety Authority (EFSA), which is no less than 5 mg of hydroxytyrosol (HT) per 20 mL of olive oil (OO) to provide protection of blood lipids from oxidative stress. Furthermore, the polyphenol intake in the OLE group was clearly higher than that of the PBO, who consumed slightly more than the instructed 20 mL.

No participants taking the PBO reported any adverse reactions and the OLE supplement was well tolerated in most participants, with the exception of one participant, who reported nausea and dropped out of the study after three days. Volunteers consistently reported that the OLE supplement had an unpleasant, bitter taste and they did not enjoy taking it.

The three-day food diaries confirmed that participant diets did not contain abnormal amounts of either polyphenol-rich foods or fish that could influence or bias the results. No changes in the food intake between groups or time points were detected.

### 2.2. RNA Quality Control

The measured RNA Integrity Number (RIN) for all samples was above 8.0, demonstrating that the RNA integrity was satisfactory for analysis. The RNA quality was evaluated again within the computer programme Affymetrix Expression Console Software (version 1.4.1, Affymetrix, Santa Clara, CA, USA) [12]. All bacterial control probes were measured in the correct order with increasing intensity, indicating that both labelling and hybridising for the arrays were accurate. A signal boxplot confirmed that the data were normally distributed.

### 2.3. PBMC Transcription Profiles

Using the Benjamin and Hochberg method of False Discovery Rate (FDR) control [13] and an adjusted *p*-value of <0.05, no genes would be considered significantly affected by the OLE supplementation in comparison to PBO when assessed using microarrays. However, using the less conservative statistical criterion of an unadjusted *p-*value <0.05, 2683 probes were differentially expressed. In addition, 1576 were upregulated and 1107 were downregulated. A less conservative *p*-value has been employed in similar human intervention studies [14,15].

Using a 1.4-fold change (FC) cut-off for expression, 27 genes were shown to be differentially expressed; these genes were used for pathway analysis. This FC cut-off has been used in previous nutrigenomic studies [14,16]. The genes were identified and classified using Gene Ontology (GO) [17,18] and listed with gene names, FC, and *p-*values.

Genes with the highest FC had large confidence intervals, emphasizing high inter-individual variance in gene expression. Genes such as *G0*/*G1 switch 2* (*G0S2*) and *chemokine* (*C-X-C motif*) *ligand 8* (*CXCL8*, subsequently referred to as *IL-8*) showed large confidence intervals compared to *EGR1*, *prostaglandin-endoperoxide synthase 2* (*prostaglandin G*/*H synthase and cyclooxygenase*) ((*PTGS2*) referred to as *COX-2*) and *heparin-binding EGF-like growth factor* (*HBEGF*) (Table 1). Changes in participant gene expression tended to show downregulation opposed to upregulation after OLE supplementation compared to PBO.

### 2.4. Gene Ontology: Gene Annotation Tool to Help Explain Relationships (GATHER) and Protein Analysis through Evolutionary Relationships (PANTHER)

Several physiological and biological processes were identified by GATHER [19] when genes with an FC > 1.4 were analysed GO indicated that OLE was having several effects on gene expression (Appendix Table A4). However, the identified pathways achieved low Bayes factors and encompassed a wide range of functions potentially regulated; therefore, further analysis was required.

**Figure 1:**
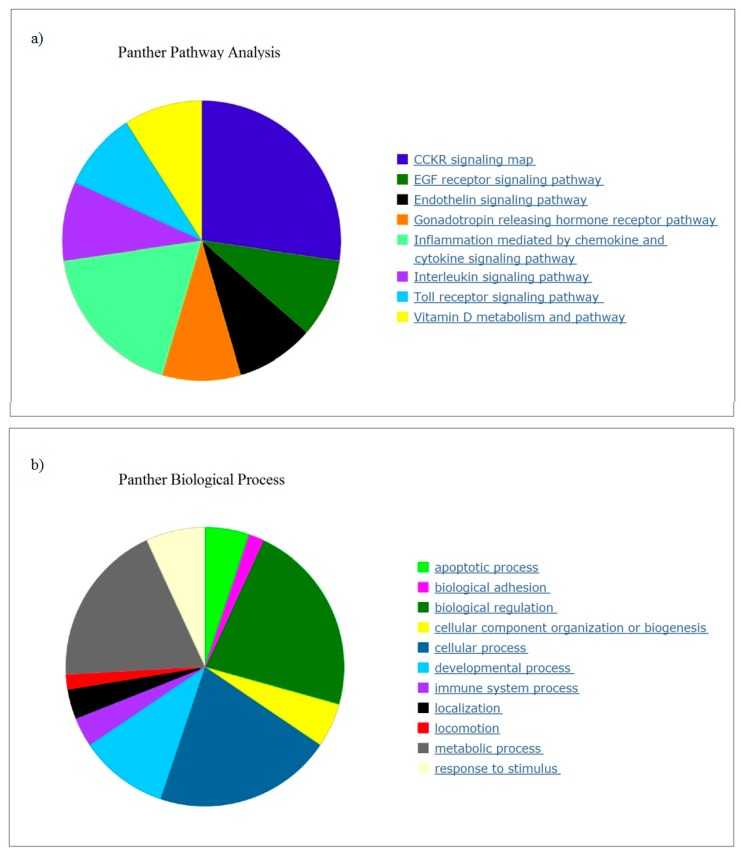
Gene classification pie charts created in PANTHER (Protein ANalysis THrough Evolutionary Relationships). (**a**) pathways; and (**b**) biological process associated with the >1.4-fold change (FC) gene expression change in response to olive leaf extract (OLE) consumption. The key names the pathways (starting from the top and moving clockwise) that have been regulated with OLE relative to placebo (PBO).

Genes with FC > 1.4 were entered into the online tool, PANTHER [20], for functional classification. The top pathway identified was cholecystokinin receptor (CCKR). Pathways related to inflammation were also highlighted, with chemokine and cytokine signalling and interleukin signalling appearing in the top altered pathways (Figure 1a). The biological processes identified by Panther confirmed a change associated with immune function; however, metabolic processes and apoptosis were the strongest relationships identified (Figure 1b).

### 2.5. Ingenuity Pathway Analysis (IPA)

To identify the biological processes altered by OLE supplementation, the genes with significant changes in expression were entered into the bioinformatics program Ingenuity Pathway Analysis, build version 346717M (QIAGEN, Redwood City, CA, USA) [21]. Using genes at an FC of >1.4 and a *p*-value of <0.05 indicated an anti-inflammatory effect with the top canonical pathways associated with cancer, inflammatory disease, and arthritis.

**Figure 2:**
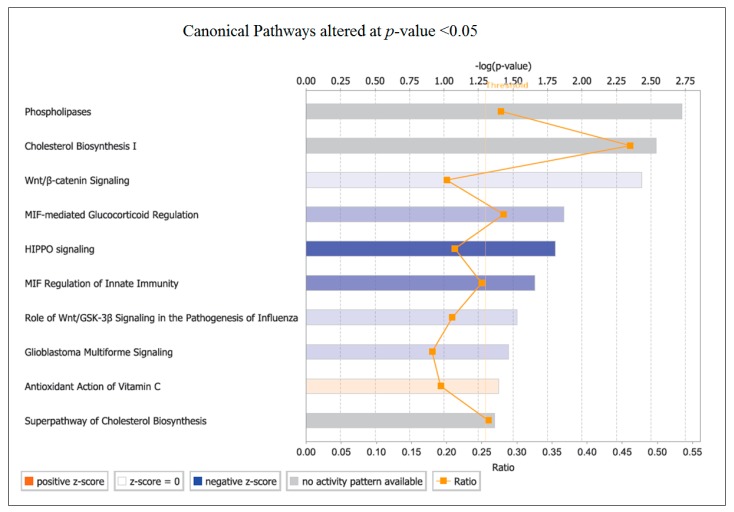
Canonical pathways affected after OLE supplementation under the criteria *p* < 0.05.

Using an FC > 1.4, the cumulative effects of small changes in gene expression are likely to have been overlooked. A further analysis was therefore undertaken including all genes that were significantly altered (*p* < 0.05. A FC of >1.4 predominantly identified genes involved downstream or at the end of pathways for inflammation. Significantly (*p* < 0.05) altered genes with a lower FC may play a pivotal role in these downstream changes. For example, the amplification of kinase cascades means that molecules upstream are likely to have a much lower FC than downstream molecules, while still exerting an important biological effect. The analysis regardless of FC identified Phospholipase (PLA; *p* = 1.89 × 10^−3^) and Cholesterol Biosynthesis (*p* = 2.89 × 10^−3^) as the top pathways (Figure 2). The top diseases and disorders identified were Cancer and Organismal Injury and Abnormalities, corresponding with the results obtained using the 1.4 FC cut-off.

**Figure 3:**
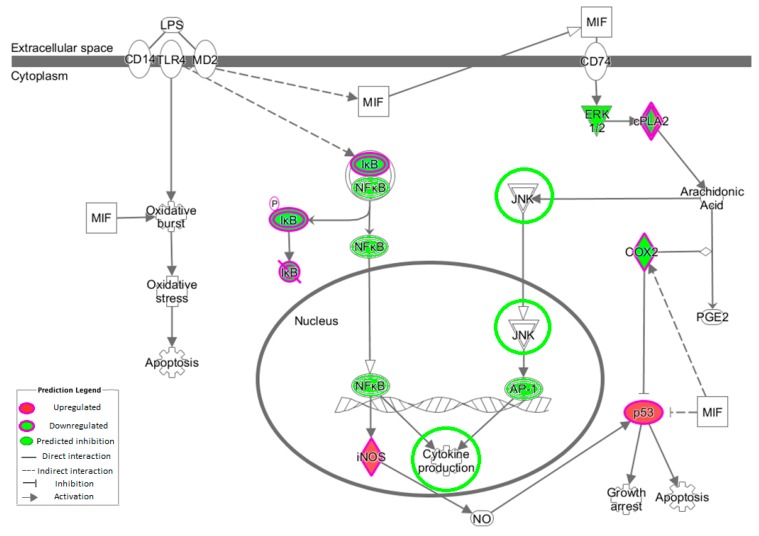
Macrophage migration inhibitory factor (MIF) regulation of innate immunity canonical pathway. Cytokine production is circled in green because there are several related cytokines, including *IL-8* (−2.4 FC) and oncostatin M (*OSM*) (−1.43 FC), which were downregulated with OLE consumption. *JNK* has been circled because of its relevance to *JUNB* regulation (downregulated in this study with −1.3 FC), which has recently been revealed to play a similar role in activating *AP1* and inflammation [22].

Phospholipases are a key component of inflammation via their release of arachidonic acid (AA), which interacts with COX-2 to produce prostaglandins (Figure 3). At >1.4 FC, Prostanoid Biosynthesis was highlighted as a key pathway; this is regulated by COX enzymes and produces an inflammatory response when activated. Metabolism of membrane lipid derivatives and cholesterol concentration interconnect and relate to the PLA pathway.

The majority of the pathways altered were downregulated (negative z-score) or had genes changed in both directions (no activity pattern predicted), although the effect of a gene being up or downregulated depends on its function within the pathway. The “Antioxidant Action of Vitamin C” pathway was upregulated (positive z-score) (Figure 2). If OLE was acting as an antioxidant, this pathway would be predicted to act in a similar way to vitamin C.

Another pathway with a predicted downregulation was the Wnt/β-catenin canonical pathway (*p* = 3.7 × 10^−3^) (Figure 2). This plays an important role in cancer progression, adipogenesis, and lipolysis.

A positive z-score (orange) indicates that gene expression is upregulated, while a negative score (blue) represents downregulated expression. The grey bars contain genes that are up and downregulated; therefore, the activity pattern cannot be determined. Ratio is the number of genes from a pathway that were altered with OLE consumption. The *p*-value for each pathway is presented on a log scale to allow easier visualisation.

#### 2.5.1. Upstream Regulators

**Figure 4:**
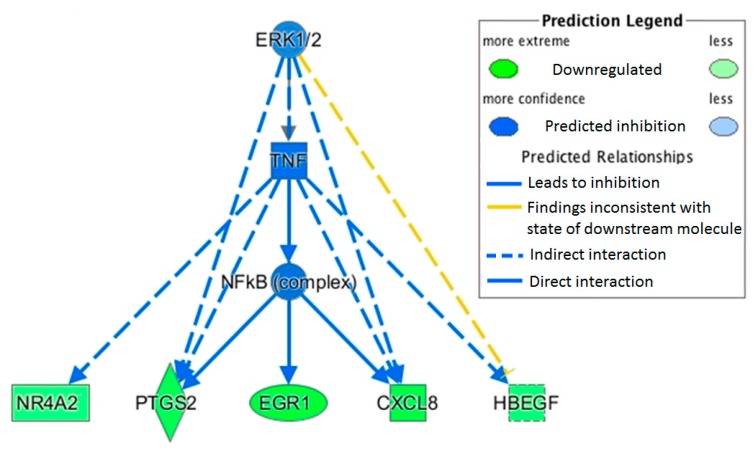
A schematic diagram of the extracellular signal–regulated kinase 1/2(ERK1/2) pathway.

Using the measured changes in gene expression, IPA can be used to elucidate the biological causes and the downstream effects on cellular and organismal biology. This analysis showed that ERK1/2 (extracellular signal–regulated kinases1/2) inhibition, via NF-κB downregulation, could result in the observed expression of several inflammatory and cancer related genes (Figure 4).

Pathways Analysis Network in IPA predicted how changes in the ERK1/2 pathway could result in the observed changes in gene expression due to OLE consumption.

#### 2.5.2. Real-Time PCR Confirmation of Array Expression

The expression levels of three genes (*EGR1*, *COX-2* and *ID3*) were evaluated with real-time PCR to validate the microarray results. An upregulated gene (*EGR1*) and a downregulated (*ID3*) were chosen to validate if the Affymetrix array had measured up and downregulated genes correctly. The expression of *COX-2* was also compared because this gene had interesting implications for inflammation. Gene expression was normalised against the housekeeping genes *GAPDH* and *ACTB* (β-actin); both of these showed consistent expression across participants, indicating that they are appropriate to use as housekeeping genes. The results measured by real-time PCR for *EGR1*, *COX-2*, and *ID3* were consistent with the microarray data (Table 2).

## 3. Discussion

To our knowledge, this is the first study that has used transcriptomics methods to analyse the gene expression changes associated with OLE consumption in humans. It is important to acknowledge that this was a preliminary study to identify where changes in gene expression are occurring. The sample size was small. Thus, the results are indicative rather than conclusive but will help guide the direction of future research. The results suggest that anti-inflammatory and cancer-related gene expression changes are associated with the consumption of OLE and could explain the health benefits claimed with traditional use. Changes in gene expression indicate that the anti-inflammatory profile involves phospholipase and inflammatory pathways. There was very little evidence that changes in gene expression relating to the phase II enzymes and the Nrf2 canonical pathway were altered by OLE consumption, although inhibition of this pathway by olive oil phenolics has been suggested as instrumental in oxidative and inflammatory protection [6,7]. Many of the downregulated genes are pivotal in inflammation and disease (*OSM*, *COX-2* and *IL-8*). Metabolic processes were also emphasized and an important lipolysis-related gene (*G0S2*) was altered by OLE consumption.

### 3.1. Gene Expression in PBMCs

PBMCs were chosen for analysis because they are easily accessible and express many genes believed to be restricted to non-blood tissues that respond to micro and macro alterations to organs, and will reflect effects of diet and nutrients [23,24]. Although mRNA expression profiles do not allow direct determination of protein function (especially those involved in phosphorylation signalling cascades), they do allow investigation of novel signalling pathways that OLE polyphenols may act through in human PBMCs. In a sample of healthy people taking a nutraceutical, changes are likely to be numerous but subtle [25], and thus it was not unexpected that the FC and significance of gene expression was not very strong. It is acknowledged that in this study, using an adjusted *p*-value cut off at 0.05, no changes in gene expressions would be deemed statistically significant. Therefore, an unadjusted *p*-value cut off at 0.05 was used. Three genes differentially expressed in response to supplementation were selected for validation with real-time PCR. One gene was significantly downregulated (*EGR1*) and one gene was significantly upregulated (*ID3*) when assessed by microarray. The third gene (*COX-2*) was selected because it plays an important role in inflammatory and cancer pathways. The expression levels between microarrays and real-time PCR for all three genes were similar, giving credibility to the use of unadjusted *p*-values for microarray analysis. All three genes used for validation play a role in cancer.

Many of the top genes downregulated in response to OLE were relevant to chronic and acute inflammation. Pathway analysis correlated the expression to inflammation and metabolic processes and identified cancer, arthritis, and adipogenesis as relevant. A recent study identified potential target genes for therapy for inflammation (*COX-2*), immune imbalance (*IL-8*), and active atherosclerosis (*OSM*) [26]. These genes were all downregulated with OLE.

### 3.2. COX-2 Expression in PBMCs

Research around the inflammatory enzyme COX-2 is extensive. This gene encoding this enzyme (*COX-2*) was downregulated in response to OLE consumption. At the time this study was completed, there were no existing studies that had demonstrated that olive polyphenols were able to regulate *COX-2* beyond post-prandial consumption in healthy humans.

Interest in the anti-inflammatory properties of oleocanthal (an olive phenolic) was motivated by a study that demonstrated its ability to decrease the COX-2 enzyme levels in vitro [27]. Expression of the gene was not investigated. It was hypothesized that oleocanthal was acting in a similar manner to ibuprofen (IB) (inhibiting the enzyme) but to gain the same effects that the recommended dose of IB has on COX-2, it has been estimated that approximately 500 g of extra virgin OO (EVOO) would need to be consumed each day [27]. This is clearly not a realistic daily dose. *COX-2* inhibition has been definitively demonstrated in cell models [28,29] and mice [30,31] treated with olive polyphenols. Cell models used polyphenol extract while the animal models have distributed it in their food with OO or refined oil, respectively.

### 3.3. IL-8 Expression in PBMCs

In this study, *IL-8* was downregulated with OLE. This corresponds to the most recent in vivo work with the same OLE supplement in humans in which reduced levels of *IL-8* were observed in blood taken from participants after a six-week intervention [9]. Ex vivo work by this group has also shown that OLE is able to downregulate *IL-8* [32]. Another study looking at overweight males found no changes to *IL-8* with OLE *IL-8* is a pro-inflammatory chemokine involved in cellular response to inflammation, tumour proliferation, and has been acknowledged to play an important role in cancer [33]. *IL-8* is a target gene of NF-κB [34], and, as mentioned above, has been identified as a potential target therapy for immune imbalance.

### 3.4. Other Genes with Inflammatory Relevance

This study measured a downregulation of transcription factor jun-B (*JUNB*) with OLE. *JUNB* is an AP1 transcription factor correlated to immune function, and recent experimental work has demonstrated its pivotal role in macrophage activation [22,35]. *JUNB* can be directly activated by NF-κB in dendritic cells with LPS stimulation [36] and is required for the full activation of IL1β and tumor necrosis factor (TNF) in macrophages stimulated by LPS [22].

Heparin binding EGF-like growth factor (*HBEGF*) was also downregulated with OLE. This growth factor is activated by the transcription factors AP1 and NF-κB and plays a role in cancer and other chronic diseases [37]. Upregulation of *HBEGF* has been observed in several cancer cells including breast (MDA-MB-231) and prostate (PC3), and it has been suggested as a potential target for treatment [38]. Its upregulation has also been correlated to NF-κB activation of inflammatory cytokines [39].

*OSM* was downregulated with OLE consumption in this study. This gene encodes the pro-inflammatory cytokine OSM, expression of which has been correlated with metastasis of cancer tumours [40,41]. In endometrial cancer, OSM was shown to activate STAT3, enhance cell migration and invasion in vitro and promote cell proliferation, growth and angiogenesis in vivo [42]. *OSM* receptor knock-out (KO) mice with cardiac disease have demonstrated improved survival and cardiac performance [43]; therefore, *OSM* could be a target for protection from cardiovascular complications.

### 3.5. NF-κB Inflammatory Pathway

The transcription factor NF-κB activates >150 genes in response to stress [44]. The NF-κB complex resides inactively within the cytoplasm due to the presence of the inhibitor molecule IκB [45]. Measuring changes in the gene expression of NF-κB is largely ineffective because it is already present and therefore does not require further protein synthesis, merely activation. The kinases inhibitor of nuclear factor κB kinase subunit (IKK)α and IKKβ phosphorylate IκB proteins, allowing NF-κB to enter the nucleus, bind to promoter regions of target genes and enhance transcription of genes [46]. The intervention work revealed no measurable change in expression of *IκB* or *NF-κB,* although there was a modest decrease in the expression of *IKKβ*. This would result in decreased phosphorylation of IκB and therefore decreased release of NF-κB. IKK can be triggered by pro-inflammatory cytokines [47]. MyD88 was also downregulated and plays an important role in the activation of NF-κB triggered by TLRs. Lastly, *NKAPL* was downregulated with OLE. An in vivo model has demonstrated that its suppression leads to downregulation of *NF-κB* via inhibiting *TNF* and *IL1β* [48].

MAPK/AP1 may also play a role in OLE inflammatory changes. In this study, *JUNB* expression was downregulated, and this plays a role in the MAPK pathway (Figure 3).

### 3.6. Lipid Metabolism and Anti-Inflammatory Gene Expression

The top canonical pathway identified by pathway analysis was PLA. Phospholipases are enzymes that hydrolyse phospholipids into fatty acids and are categorized by the type of reaction they catalyse [49]. The genes involved in changes with OLE were predominantly associated with phospholipase A2 (PLA_2_) and then from within Phospholipase C (PLC). The PLC enzymes play an important role in cell signalling, proliferation and differentiation [50].

PLA_2_ plays an important role in inflammation via the release of arachidonic acid (AA) from the phospholipid membrane [51]. AA is transformed into prostaglandin (PGE2), which is converted by COXs to eicosanoids (including prostaglandins and leukotrienes), which influence both innate and adaptive immunity [52,53]. The downregulation of *COX-2* observed in this study could restrict prostanoid (prostaglandin, thromboxane and prostacyclin) production from AA. Many diseases and their related complications have been correlated to PLA_2_ upregulation, including cardiovascular complications, atherosclerosis, vascular inflammation, cancer and obesity [53,54,55,56]. Inhibition of this pathway would partially explain the downregulation of inflammatory genes in this study.

*G0S2* showed the strongest FC in expression after OLE consumption compared to all the other regulated genes, which is a novel finding. This gene has recently been highlighted for its ability to inhibit adipose triglyceride lipase (ATGL) which is key for the mobilization of triacylglycerol stores [57]. We are not aware of any studies that have reported or investigated changes in *G0S2* with polyphenols. *G0S2* KO in high fat diet-fed mice protects from obesity and insulin resistance, and increases thermogenesis [58,59,60]. Zhang et al. [59] measured an increase in ketogenesis (measured by plasma ketones). Silencing of *G0S2* prevented adipocytes from differentiation, while overexpression activated progression from pre-adipocytes to mature adipocytes in vitro and in vivo. *G0S2* KO reduced fat mass, lipid droplet size and the adipogenesis pathway in mice [57]. This suggests that *G0S2* is a key regulator of energy homeostasis, mediating lipolysis and fatty acid oxidation, and a downregulation of this gene could be beneficial in the treatment of both obesity and obesity-related diseases.

### 3.7. Gene Expression Change and Health Outcomes

The anti-inflammatory gene expression demonstrates that changes are not merely due to anti-oxidising capacity as was historically hypothesised. Downregulating the AA and NF-κB pathways could explain many of the health benefits attributed to OLE with traditional use. These include pain, inflammatory related diseases such as cancer, diabetes, cardiovascular disease, and an improved acute immune response.

Chronic inflammation is linked to many diseases including cancer and cardiovascular disease. *COX-2* is a strong driver of inflammation, and, therefore, has been an important area of focus [61]. The AA pathway and PGE2 production have been correlated with cancer and are also known as the “inflammogenesis of cancer” [53]. Regular intake of *COX-2* inhibitors such as aspirin has been associated with a reduced risk of death due to cancers including prostate and breast [61,62,63]. Upregulation of *COX-2* is found in skin, breast, prostate, bladder and pancreatic carcinomas [64].

Inhibiting *COX*-2 and thereby reducing prostaglandins could also explain improvements in Blood Pressure (BP) measured in humans after OLE consumption [9]. HT administered to rats was able to reduce thromboxane B_2_ and platelet aggregation in blood [65]. In a model using hypertensive rats, COX-2 inhibitors reduced Prostaglandin F2α (PGF2α) expression, improved endothelial relaxation and reduced BP [66]. In humans, intake of high phenolic enriched OO has also shown improved BP with reduced chemokine (C-X-C motif) receptor 2 (*IL8RA*) and other genes related to BP [67]. This study explained that the reduction in *IL8RA* could influence the renin–angiotensin–aldosterone system II, which plays an important role in blood pressure and thereby hypertension. Although *IL8RA* was not identified in our study, it is a specific receptor for *IL-8*. In studies investigating postprandial effects of olive oil phenolic consumption in humans, the anti-inflammatory profile was evident but an upregulation in cholesterol efflux was also demonstrated [68,69]. Cholesterol accumulation in immune cells leads to inflammatory responses, which, in turn, can lead to decreased cholesterol efflux and further inflammation [70]. A reduction in oxidised cholesterol could help explain the anti-inflammatory profile measured with OLE in our study.

In downregulating acute inflammation, OLE would be hypothesized to compromise the immune system and conceivably facilitate infection. More recently, it has been suggested that bacteria may benefit from and further stimulate PGE2 synthesis in order to compromise the gut and lung linings of the host [71]. In human macrophages, an inhibition of COX-2 reduced H5N1 infected pro-inflammatory response and virus replication, the work indicated the virus required COX-2 pathway activation in order to replicate [72]. IL-8 production is also activated by influenza [73]. A downregulation of inflammatory related genes as observed in this study with OLE could improve immune function and defence against pathogen attack.

This study demonstrated downregulation in *G0S2* with OLE supplementation. If *G0S2* was able to downregulate free fatty acid (FFA) or adipogenesis, this could contribute to the reduced inflammatory profile measured in participants taking OLE in this study.

## 4. Materials and Methods

### 4.1. Study Design

**Figure A1:**
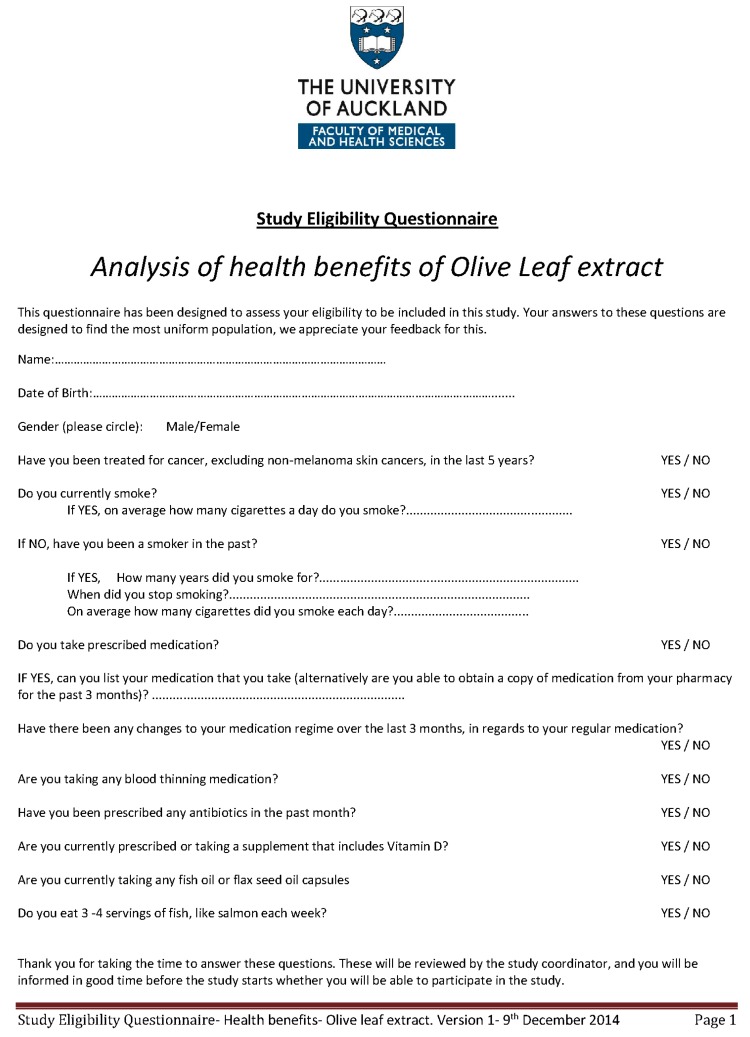
Eligibility question used to determine selection of the healthy study participants.

The study was a parallel double-blind randomised controlled trial conducted according to the ethical guidelines laid down by the Declaration of Helsinki 1975, and approved by the New Zealand Health and Disability Ethics Committees (15/NTB/27). The study was registered with the Australian New Zealand Clinical Trials Registry (ACTRN12615001021561). An 8-week intervention time was based on a previous 6-week intervention study measuring changes in BP after supplementation with the same OLE product [9]. A more recent study looking at polyphenols in the diet using a parallel design also used 8 weeks to successfully detect changes in inflammation [74]. OLE and Mediterranean Diet benefits to health are correlated to long-term exposure; therefore, this study was looking for an alteration in gene expression that was not merely a post-prandial effect. Thirty-three male subjects were given either a liquid OLE supplement or PBO (Appendix Table A2). Exclusion criteria included the consumption of anti-inflammatories (such as low dose aspirin), antioxidant supplements, cholesterol or blood pressure medicine, and antibiotics in the past month (Appendix Figure A1). Participants were instructed to take 10 mL of their supplement twice a day with meals for 8 weeks. Measuring spoons were provided. Participants attended a clinical visit at the beginning of the trial where they gave a fasted blood sample and completed their initial Food diary. After the 8-week intervention, participants returned for their second visit where they gave a fasted blood sample. A second food diary was handed in at this appointment. An 8–10 mL blood sample was collected in ethylenediaminetetraacetic acid (EDTA) tubes at the Faculty of Medical and Health Sciences, Grafton campus, The University of Auckland by a registered nurse.

### 4.2. Test Product

“Olive Leaf Extract Extra Strength” is a commercially available OLE liquid concentrate manufactured and bottled by Comvita New Zealand Limited (Paengaroa, Te Puke, New Zealand) (Appendix Table A2). Both OLE and PBO were analysed by high performance liquid chromatography (HPLC), which allowed the type and concentrations of polyphenols to be determined. This showed that the PBO contained no polyphenols (Appendix Table A3). A 10 mL dose twice a day provided 121.8 mg of Oleuropein and 6.4 mg of HT daily. Tests have shown that even at high concentrations, OLE is not genotoxic [75].

### 4.3. Statistical Analysis and Sample Size

There was no published literature on OLE and transcriptomic outcomes in humans at the time this research was undertaken; therefore, OO phenolic studies in humans were utilized to inform statistical decisions.

Standard deviation and differential expression of the *JUN* gene in healthy volunteers was taken from a previous study looking at changes with OO phenolics [76]. This standard deviation was used to calculate a total sample of 32 participants allowing >80% power to detect a significant difference in *JUN* gene expression between intervention groups assuming a dropout rate of 10% and a 2-sided type error of 0.05. The linear models for microarray analysis (Limma) model [77] was used for analysing gene expression pre and post intervention, paired by participant.

### 4.4. Dietary Assessment

All volunteers were asked to complete a diet and activity profile, using the University of Auckland standard 3-day food and activity diary.

The food intake diaries collected pre and post-supplementation were analysed using Foodworks Professional 2012 (Xyris Software (Australia) Pty Ltd., Brisbane, Autralia) and the New Zealand food composition tables. These were used to estimate changes that could impact the gene expression measured in PBMCs, such as high alcohol or polyphenol consumption over the days before the clinical visit. The diary was also used to measure any changes in the diet that may have occurred after taking the supplement.

### 4.5. Compliance

Volunteers from both intervention arms were contacted fortnightly by the coordinating investigator to confirm that the supplement was being taken and there were no problems. The instructions were to take 20 mL per day of the cardiovascular strength OLE, delivering 121.8 mg of oleuropein and 6.4 mg HT. Remaining supplement was returned at the end of the study, and this was used to estimate the amount of supplement taken.

### 4.6. Gene Expression Arrays

PBMCs were extracted from the blood samples by Ficoll–Paque density gradient centrifugation (Ficoll–PaqueTM Premium, Global Science and Technology, Auckland, New Zealand) and stored until RNA extraction.

All RNA samples were extracted from PBMCs in batches using the RNeasy Plus Mini Kit (QIAGEN, Victoria, Australia) following the manufacturer’s recommended protocol.

RNA was quantified using the NanoDrop^®^ ND-1000 spectrophotometer (Thermo Fisher Scientific, Wilmington, DE, USA) and quality tested using the Bioanalyzer (2100 Bioanalyzer, Aligent, Santa Clara, CA, USA) analysis. The RNA was then diluted to a concentration of 100 ng/µL.

The data discussed in this publication have been deposited in NCBI’s Gene Expression Omnibus [78] and are accessible through GEO Series accession number GSE87300 (https://www.ncbi.nlm.nih.gov/geo/query/acc.cgi?acc=GSExxx).

### 4.7. Microarray Analysis

The RNA samples were sent to the Ramaciotti Centre, Randwick, Australia for analysis. An Affymetrix PrimeView™ gene array was used to quantify gene expression, analysed by the GeneChip Scanner 3000 7G (Affymetrix).

### 4.8. Quality Control of Arrays

Quality assessment of the microarrays was completed using Affymetrix Expression Console (Thermo Fisher Scientific) and the BioConductor package “affy” [79] in R, version 3.1.3 [80].

### 4.9. Differential Gene Expression

To detect changes after OLE intervention, the full set of OLE volunteer arrays were compared to the paired baseline array. The arrays from the PBO volunteer group were also compared to their baseline array. Changes in the PBO arm arrays were used to control for the natural fluctuation in gene expression that would also be expected within the OLE arm. A mathematical linear model was used to calculate differences between the intervention groups and timepoints. The age variation was also accounted for in the linear model.

The package “ggplot” in R was used to visualize the expression of specific probe sets or genes of interest pre and post-supplementation and between treatment groups.

### 4.10. Gene Ontology

Gene Ontology (GO) analysis was carried out with PANTHER to elucidate biological process, molecular function and the protein class [17]. The Gene Ontology project is a bioinformatics initiative encompassing knowledge of how genes encode biological functions at the molecular, cellular and tissue system levels.

Significantly altered genes were also entered into GATHER [19], which creates a Bayes factor. This is a measure of the strength of the evidence supporting an association of an annotation with the gene list submitted [19]. Higher Bayes factors indicate stronger evidence that the annotation is relevant to the genes of interest [81]. A positive Bayes factor indicates that the evidence supports the hypothesis that the annotation is more related to the genes of interest than other genes in the genome. This can create bias when looking for pathway and gene group changes because the strength of the Bayes factor is based upon the ratio of genes altered from a pathway to the genes altered. GATHER gene expression analysis is a preliminary analysis specialising in identifying the potential pathways of interest based on the genes found to be significantly affected.

### 4.11. Ingenuity Pathway Analysis (IPA)

Pathway analysis of the differentially expressed genes was performed using IPA software (2000–2016, QIAGEN, Redwood City, CA, USA). This program constructs gene interactions based on the regularly updated Ingenuity Knowledge Base (http://www.ingenuity.com). This program is also able to predict in which direction these changes are likely to impact: inhibitory or activating. The statistical modelling behind IPA uses the Fisher exact test [82] to determine significant pathways from the input gene list.

### 4.12. Gene Expression Validation with Real-Time PCR

To confirm the microarray results, TaqMan real-time PCR analysis was performed for *EGR1*, *ID3* and *COX-2* following the manufacturer’s protocol. Two housekeeping genes (*GAPDH* and β-actin) were used to control for expression.

### 4.13. Statistical Analysis of Real-Time PCR

Gene expression levels were calculated with the equation: 2^ΔΔ*C*t^ [83]. The gene expression results for housekeeping genes were combined allowing for greater accuracy [84]. An absolute FC was calculated to allow ease of analysis, and this was calculated with the equation: −2^^Log2(FC)^. An FC greater than 1 was classified as an upregulation of gene expression and less than 1 was classified as downregulation of gene expression. As with the statistical analysis of the microarray data, the PBO was used to adjust for the FC in OLE participants. In order to calculate the FC differences between OLE and PBO, data for the participants treated with OLE was adjusted with participants treated with PBO following calibration, using a modified formula from Section 4.9.

*(OLE_T2 calibrated with OLE_T1) − (PBO_T2 calibrated with PBO_T1)*

If the range of standard error of the mean intersected with 1 or −1, then the expression was considered non-significant.

A Students *t*-test was used to calculate the differences in gene expression between the OLE treatment and PBO treatment. Two tailed *p*-values were calculated EGR1 *p* = 0.025, COX-2 *p* = 0.016 and ID3 *p* = 0.023.

## 5. Conclusions

To conclude, the gene expression profile observed in this study with OLE may explain health benefits described in previous studies. Improvements in BP [9] and insulin sensitivity [10] measured in humans after the consumption of OLE relate to regulation of phospholipase metabolic and inflammatory pathways. Anti-inflammatory gene expression in humans could explain the claimed health outcomes for cold and influenzas, and chronic anti-inflammatory alterations to gene expression could explain reduced prevalence of cardiovascular disease and cancers.

Literature supports olive polyphenol ability to improve health outcomes related to inflammation. What bioactive components of the OLE are interacting with the inflammatory pathways deserves further analysis. The anti-inflammatory expression induced with OLE could be a result of manipulation of lipid metabolism or vice versa, but further investigation is required.

## Figures and Tables

**Table 1:** Fold change (FC) in gene expression after olive leaf extract (OLE) supplementation relative to placebo (PBO) with *p*-values.

Gene Symbol	Gene Name	FC	*p*-Value
*G0S2*	G0/G1 switch 2	−3.121	9.65 × 10^−3^
*EGR1*	early growth response 1	−2.54	1.81 × 10^−2^
*CXCL8 (IL-8)*	chemokine (C-X-C motif) ligand 8	−2.397	3.93 × 10^−2^
*COX-2 (PTGS2)*	prostaglandin-endoperoxide synthase 2	−2.241	7.49 × 10^−3^
*EGR2*	early growth response 2	−1.978	2.77 × 10^−2^
*KLF3*	Kruppel-like factor 3 (basic)	−1.858	1.08 × 10^−2^
*HBEGF*	heparin-binding EGF (epidermal growth factor) -like growth factor	−1.786	1.30 × 10^−4^
*NR4A2*	nuclear receptor subfamily 4, group A, member 2	−1.625	3.29 × 10^−2^
*NDC80*	NDC80 kinetochore complex component	−1.599	3.48 × 10^−4^
*MXD1*	MAX (myc-associated factor X) dimerization protein 1	−1.543	3.36 × 10^−2^
*NCAPG*	non-SMC (Structural Maintenance of Chromosomes) condensin I complex, subunit G	−1.526	2.67 × 10^−2^
*OSM*	oncostatin M	−1.428	2.98 × 10^−2^
*NAP1L3*	nucleosome assembly protein 1-like 3	1.463	8.88 × 10^−3^
*MIER3*	mesoderm induction early response 1, family member 3	1.47	6.14 × 10^−4^
*ID3*	inhibitor of DNA binding 3, dominant negative helix-loop-helix protein	1.483	9.27× 10^−3^

**Table 2:** Real-time PCR confirmation of the gene expression measured by Affymetrix arrays. Differential expression of *EGR1*, *COX-2* and *ID3* following the eight-week supplementation with OLE relative to PBO. Two housekeeping genes were combined (*GAPDH* and β-actin) for reference. FC is relative to PBO.

	Gene Expression FC		
	*EGR1*	*COX-2*	*ID3*
Real-time PCR	−2.18052	−1.73258	1.801411
Standard deviation	0.141586	0.145314	0.10132
Affymetrix Array	−2.54	−2.24	1.48
*p*-Value	1.81 × 10^−2^	7.49 × 10^−3^	9.27 × 10^−3^

## Abbreviations

AA	Arachidonic acid
*AP1*	Activator protein 1
*COX-2*	Prostaglandin-endoperoxide synthase 2 or cyclooxygenase 2
*EGR1*	*Early growth response 1*
ERK	Extracellular signal-regulated kinase
FC	Fold change
FFA	Free fatty acid
GO	Gene ontology
*G0S2*	*G0/G2 switch gene 2*
*HBEGF*	*Heparin-binding EGF-like growth factor*
HT	Hydroxytyrosol
*ID3*	Inhibitor of DNA binding 3, dominant negative helix-loop-helix protein
KO	Knock out
MIF	Macrophage migration inhibitory factor
NF-κB	Nuclear factor kappa-light-chain-enhancer of activated B cells
OLE	Olive leaf extract
OO, VOO, EVOO	Olive oil, virgin olive oil, extra virgin olive oil
*OSM*	*Oncostatin M*
PGE2	prostaglandin
PBO	Placebo
PLA, PLA_2_, PLC, sPLA, cPLA	Phospholipase, phospholipase A2, phospholipase C, secreted phospholipase, cystolic phospholipase

## Appendix A

**Table A1:** Characteristics of study participants pre- and post-supplementation.

Measurement	Olive Leaf Extract Group	*n*	Placebo Group	*n*
Age	32 years (mean)		34.5 years (mean)	
	20–29	9	20–29	7
	30–39	3	30–39	2
	40–49	1	40–49	2
	>50	2	>50	2
Smoking Status				
	Never	12	Never	9
	Former	3	Former	4
	Current	0	Current	0
BMI Pre-supplement	24.93 kg/m^2^ (mean)		24.21 kg/m^2^ (mean)	
	<20	0	<20	0
	≥20–≤25	10	≥20–≤25	9
	>25–≤30	3	>25–≤30	4
	>30	2	>30	1
BMI Post-supplement	24.26 kg/m^2^ (mean)		24.07 kg/m^2^ (mean)	
	<20	0	<20	0
	≥20–≤25	10	≥20–≤25	9
	>25–≤30	4	>25–≤30	4
	>30	1	>30	1

**Table A2:** Ingredients for placebo and OLE supplement.

Placebo-Recipe	Olive Leaf Extract
30 L Glycerol	15 L Vegetable glycerol (bulking agent)
15 g Sucrose octa acetate added to 30 L water	15 L *Olea europaeo* (Olive) Leaf extract (water, olive leaf solids) equivalent to 15 g fresh leaf (5 g fresh leaf per 5 mL dose)
98 g Caramel colour	
0.9 g Damascenone	
0.075 g Guaiacol	
0.075 g Eugenol	

**Table A3:** Phenolic content in Comvita OLE (HPLC performed by Alistair Binney from the Comvita team. The placebo was also run for quality control and no polyphenols were detected).

Phenolic Content	mg/mL	Dose (mg) 20 mL
Oleuropein	6.09	121.8
Oleoside	0.81	16.2
Hydroxytyrosol	0.32	6.4
Luteolin 7-*O*-glucoside	0.1	2
Tyrosol	0.07	1.4
Vanillin Acid	0.03	0.6
Apigenin-7-*O*-glucoside	0.02	0.4
Caffeic Acid	0.02	0.4
Verbascoside	0.01	0.2
Vanillin	0.01	0.2
Rutin	0.01	0.2
Diosmetin	0.01	0.2

**Table A4:** Results generated in Gene Annotation Tool to Help Explain Relationships (GATHER) using the Gene ontology (GO) database.

Gene Ontology at >1.4 FC	Number of Genes	*p-*Value	Bayes Factor
Regulation of physiological process	13	<0.0001	10
Regulation of biological process	13	<0.0001	8
Development	9	0.0002	5
Regulation of metabolism	9	0.0004	4
Cyclooxygenase pathway	1	0.0008	4
Regulation of transcription, DNA-dependent	8	0.001	3
Transcription, DNA-dependent	8	0.001	3
Regulation of transcription	8	0.002	3
